# Is there a close association of depression with either constipation or dysosmia in Parkinson’s disease?

**DOI:** 10.1038/s41598-020-72381-0

**Published:** 2020-09-23

**Authors:** Ting-Ya Chang, Yi-Huei Chen, Ming-Hong Chang, Ching-Heng Lin

**Affiliations:** 1 Department of Neurology, Taichung Veterans General Hospital, Taichung, Taiwan; 2 Department of Medical Education and Research, Taichung Veterans General Hospital, Taichung, Taiwan

**Keywords:** Neuroscience, Neurology

## Abstract

A possible association between depression and either the severity of constipation or dysosmia in Parkinson’s disease (PD) patients was investigated in this cross-sectional study. One-hundred six patients who had the history of PD for less than 5 years were recruited. Depression was measured using the Beck Depression Inventory-II (BDI-II), and our patients were divided into depressive and non-depressive groups (DP: BDI-II ≥ 14; n = 22 and NDP: BDI-II < 14; n = 84). Olfactory dysfunction was assessed by the University of Pennsylvania Smell Identification Test (UPSIT). Constipation severity was defined by stool softener dosage and amount. Statistical analyses with one-tailed T- or chi-squared test, odds ratios (OR), and beta-coefficient were used to determine significant differences. Total scores based on the Unified Parkinson’s Disease Rating Scale (UPDRS) were significantly higher in the DP group. A significant relationship was observed between PD patients with depression and severe constipation; PD patients with depression were more likely to present with severe constipation (OR 5.81; 95% CI 1.24–27.29, p = 0.026, adjusted for age and gender); but the significance became marginal after adjusted for age, gender and UPDRS part 3 (OR 4.46, 95% CI 0.93–21.33; p = 0.061). However, no association between olfactory dysfunction and depression was detected. There were significant positive correlations between BDI-II scores and severe constipation (β ± SE 7.65 ± 2.02; p =  < 0.001, adjusted for age and gender; β ± SE 7.06 ± 2.04; p = 0.001, adjusted for age, gender, and UPDRS-3). Besides, we detected a marginally significant correlation that PD patients with higher BDI-II scores tended to present more severe motor symptoms. Olfactory dysfunction seemed to be less relevant to BDI-II scores. Based on our findings, we speculate that depression may be more closely related to brainstem nuclei than to the limbic pathway.

## Introduction

Parkinson’s disease (PD) is the second most common neurodegenerative disease, and patients present with dominant motor symptoms and a wide range of non-motor symptoms. Misfolding, aggregation, and propagation of the alpha synuclein protein (α-syn) in the central nerve system is the main pathogenesis of PD^1^. Braak et al. established the framework of α-syn propagation based on pathological evidence in which inclusions initially appear in the olfactory bulb and dorsal motor nucleus of the vagus (DMV) in the lower brain stem^2,3^. Along with the discovery that the terminals of the olfactory nerve and enteric plexus of the gastrointestinal tracts are the early sites of Lewy pathology, the dual-hit hypothesis was proposed in which it was postulated that α-syn has prion-like properties and spreads with a peripheral–to–central pattern^4^.

Depression is one of the most common nonmotor symptoms in PD, which usually worsens during “off” periods^5,6^. The mechanism of depression in PD is not clear, and the current hypothesis includes changes of neurotransmitters (such as serotonin, dopamine, noradrenaline, acetylcholine) and lesions of fronto-subcortical circuits^7^. The peripheral–to–central α-syn propagation describes PD as a multisystem disorder, and its clinical manifestations (such as depression) may be associated with the evolution of anatomical alterations and neurochemical imbalances in brainstem nuclei and cortex^8^. According to the hypothesized α-syn spreading pattern, the DMV is connected to the raphe nuclei and locus coeruleus (LC), which are connected to the substantia nigra. The olfactory bulb is also connected to the LC and substantia nigra^1^. Lewy body inclusions in the LC, raphe nucleus, hippocampal region, and cerebral cortex may be related to depression in PD^9^.

Lewy body pathology in brainstem nuclei possibly plays an important role in the pathogenesis of depression in PD patients. Therefore, we hypothesized that the degree of depression in PD patients may be closely related to the severity of constipation, which represents the spreading of α-syn from the gastrointestinal tract to the DMV in brainstem, rather than olfactory dysfunction, reflecting the involvement of the olfactory bulbs. In the present study, we hope to investigate whether depression in PD patients is closely related to either constipation or dysosmia.

## Methods

This study was approved by IRB (No.CE16171B) at Taichung Veterans General Hospital. All methods were performed in accordance with the Declaration of Helsinki guidelines and hospital regulations. Informed consent was obtained from all subjects and their informants before study participation.

### Study design, data sources and population

Patients with PD for less than 5 years were recruited from 2017 to 2018 at Taichung Veteran General Hospital. A PD diagnosis was established according to United Kingdom Parkinson’s Disease Society Brain Bank Criteria. Patients who were diagnosed with major depressive disorder or other psychiatric diseases previously were excluded. Demographic variables, including age and gender, were recorded for all participants. PD severity was rated with the Unified Parkinson’s Disease Rating Scale (UPDRS)^10^. The Mini-Mental State Examination (MMSE) was also administered. Patients were evaluated by well-trained nurses and neurologists at our hospital.

### Depression assessment (dependent variable)

We used the Beck Depression Inventory-II (BDI-II) to evaluate the severity of depression. Patients were under “on-status” conditions at the time of BDI II testing in our outpatient clinic. A BDI-II score of 0 to13 indicates minimal depression, 14 to 19 mild, 20 to 28 moderate, and 29 to 63 indicates severe depression. The patients were divided into two groups based on BDI-II scores: (1) the depressive group (DP group, BDI-II ≥ 14) and (2) the non-depressive group (NDP group, BDI-II < 14).

### Olfactory function assessment (independent variable)

Olfactory dysfunction was evaluated by the University of Pennsylvania Smell Identification Test (UPSIT, traditional Chinese version)^11,12^. This standardized test uses 40 microencapsulated odorants released by scratching the standardized odor impregnated test booklets. Patients were asked to identify the odor from four multiple choice questions provided for each question. This test has a total score of 40. After completing this test, our patients were categorized into four groups based on their scores: (1) normal (male: 34–40; female: 35–40);(2) mild hyposmia (male: 30–33; female 31–34); (3) moderate hyposmia (male: 26–29; female: 26–30); (4) severe hyposmia (male and female: 19–25); and (5) total anosmia (male and female: 0–18). The subjects were further grouped as mild-moderate hyposmia, severe hyposmia, and total anosmia^11–13^.

### Constipation assessment (independent variable)

We diagnosed patients with constipation using the Rome IV Diagnostic Criteria for functional constipation. We defined constipation severity by the dosage and intensity of stool softeners: (1) grade 0 indicated no constipation; (2) grade 1 indicated mild constipation but no laxatives or Sennoside A + B 12 mg only; (3) grade 2 indicated Sennoside A + B 24 mg or two kinds of laxatives (such as magnesium oxide, Bisacodyl, Conslife [Bisacodyl 2 mg + sennosides 10 mg + dioctyl sodium sulfosuccinate 20 mg]); (4)grade 3 indicated Sennoside A + B (36 mg) or more than three kinds of laxatives. We excluded other known secondary causes of constipation, such as colon cancer, inflammatory bowel disease, extra-intestinal mass, or surgery-related conditions.

### Statistics

For the DP and NDP groups, we used the Student’s t- and chi-squared tests to compare their age, gender, dysosmia severity (mild–moderate, severe, total anosmia), constipation severity (grades 0–3), MMSE scores, UPDRS total (UPDRS T), and part 3 (UPDRS 3, motor function) between DP and NDP groups. Odds ratios (OR) were calculated to quantify the association between dysosmia and depression, and the association between constipation and depression, using a multiple variable logistic regression analysis after adjusting for age, gender, and UPDRS 3.

Considering that most of our PD patients were not diagnosed as major depressive disorder or other mood disorders, fewer patients were categorized into the depressive group with BDI-II scores higher than 13. Therefore, we wanted to test the correlation between the BDI-II scores (continuous variable) and the two independent variables. A beta-coefficient was calculated for the correlation between dysosmia and BDI-II scores, and the correlation between constipation and BDI-II scores, using the linear regression analysis after adjusting for age, gender, and UPDRS 3.

All statistical tests were two-sided, conducted at a significance level of 0.05, and reported using p-value and/or 95% confidence intervals (CIs). Statistical analyses were performed with SAS software version 9.4 (SAS Institute, Cary, NC, USA).

## Results

### Clinical characteristics of DP and NDP groups

We initially enrolled 108 patients diagnosed with PD for less than 5 years at our hospital during the period from 2017 to 2018; however, we excluded 2 patients with extremely high BDI-II scores over the outlier level. Finally, 106 PD patients were entered into our study and they were divided into two groups based on scores BDI-II scores: (1) the DP group (BDI-II ≥ 14) and (2) the NDP group (BDI-II < 14). (Table 1). Twenty-two and 84 patients were assigned to the DP and NDP groups, respectively. The mean ages were 62.4 ± 9.8 years in the DP group and 65.1 ± 8.5 years in the NDP group. There was no significant difference in the MMSE scores between the two groups. The total scores of UPDRS were significantly higher in the DP group (61.4 ± 31.2 versus 43.5 ± 20.9; p-value = 0.020), and there was also a tendency toward higher UPDRS part 3 scores (motor function) in the DP group (34.4 ± 21.1 versus 27.4 ± 13.8; p-value = 0.160). There was no significant difference regarding olfactory dysfunction and constipation severities between the two groups before the adjustment, but a larger proportion of severe constipation (grade 3) was noted in the DP group (40.9% and 21.4%, DP and NDP group, respectively). To account for antidepressant-related constipation, we reviewed their medical records. Three of 22 patients in the DP group took antidepressants at the time of the assessment. All of them took 25 mg agomelatine at night under the indication of depression or insomnia (see Supplementary Table S1 online).

**Table 1:** Clinical characteristics of PD patients grouping by BDI-II.

Variable	Total n = 106		NDP group (BDI-II < 14) n = 84		DP group^b^ (BDI-II ≥ 14) n = 22		P-value
	n	(%)	n	(%)	n	(%)	
Age, years (mean ± SD)	64.5 ± 8.8		65.1 ± 8.5		62.4 ± 9.8		0.202
**Gender**							0.730
Female	40	(37.7)	31	(36.9)	9	(40.9)	
Male	66	(62.3)	53	(63.1)	13	(59.1)	
No. of correct detection in UPIST (mean ± SD)	17.8 ± 6.7		17.6 ± 6.6		18.5 ± 7.3		0.585
**UPSIT category**							0.414
Mild-moderate microsmia^a^	15	(14.2)	10	(11.9)	5	(22.7)	
Severe microsmia	35	(33.0)	29	(34.5)	6	(27.3)	
Total anosmia	56	(52.8)	45	(53.6)	11	(50.0)	
**Severity of constipation**							0.295
0	29	(27.4)	25	(29.8)	4	(18.2)	
1	29	(27.4)	24	(28.6)	5	(22.7)	
2	21	(19.8)	17	(20.2)	4	(18.2)	
3	27	(25.5)	18	(21.4)	9	(40.9)	
MMSE (mean ± SD)	26.9 ± 4.6		26.9 ± 4.6		27.1 ± 4.7		0.872
UPDRS T (mean ± SD)	47.1 ± 24.2		43.5 ± 20.9		61.4 ± 31.2		0.020
UPDRS 3 (mean ± SD)	28.8 ± 15.7		27.4 ± 13.8		34.4 ± 21.1		0.160

*NDP group* non-depressive group, *DP group* depressive group, *BDI* Beck Depression Inventory, *UPDRS* Unified Parkinson's Disease Rating Scale, *MMSE* Mini-Mental State Examination, *UPSIT* = University of Pennsylvania Smell Identification Test.

^a^Only 3 patients have mild microsmia, so we combined them in to moderate microsmia (n = 12) group.

^b^In the depressive group, only three patients taking antidepressants (all of them took agomelatine 25 mg at night).

### The influence of dysosmia and constipation to depression in PD

**Figure 1:**
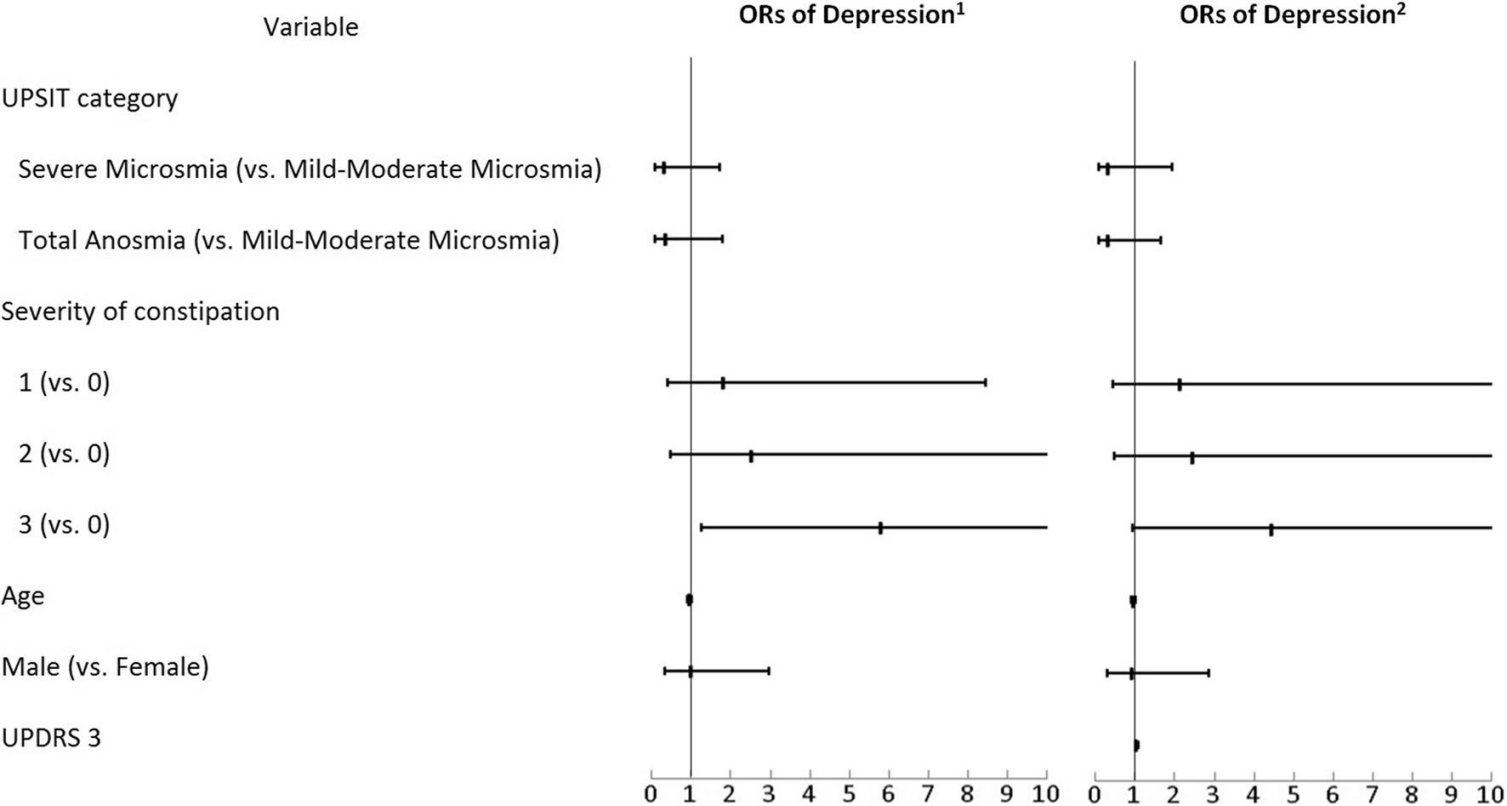
Adjusted Odds ratio of depression associated with dysosmia and constipation. ^1^Adjusted for age and gender.^2^Adjusted for age, gender and UPDRS 3. *UPDRS* Unified Parkinson's Disease Rating Scale, *UPSIT* University of Pennsylvania Smell Identification Test.

A multiple variable logistic regression model was constructed to examine the association between dysosmia and depression, and the association between constipation and depression. We adjusted the results by age, gender, and UPDRS-3. The results are shown in Table 2 and Fig. 1. We detected a significant association between PD patients with depression and severe constipation (grade 3) (OR 5.81; 95% CI 1.24–27.29, p = 0.026, adjusted for age and gender), but a marginal significance after being adjusted for age, gender and UPDRS part 3 (OR 4.46, 95% CI 0.93–21.33; p = 0.061). No association between the severity of olfactory dysfunction and depression was detected.

**Table 2:** Adjusted odds ratio of depression associated with dysosmia and constipation.

Variable	Adjusted odds ratio^a^	95% CI	P-value	Adjusted odds ratio^b^	95% CI	P-value
**UPSIT category**						
Mild-moderate microsmia	1.00	–	–	1.00	–	–
Severe microsmia	0.32	(0.06–1.72)	0.185	0.33	(0.06–1.92)	0.217
Total anosmia	0.35	(0.07–1.77)	0.204	0.32	(0.06–1.66)	0.174
**Severity of constipation**						
0	1.00	–	–	1.00	–	–
1	1.82	(0.39–8.44)	0.446	2.15	(0.45–10.38)	0.340
2	2.53	(0.47–13.58)	0.278	2.47	(0.46–13.41)	0.294
3	5.81	(1.24–27.29)	0.026	4.46	(0.93–21.33)	0.061
Age	0.96	(0.90–1.02)	0.222	0.96	(0.90–1.02)	0.191
**Gender**						
Female	1.00	–	–	1.00	–	–
Male	0.99	(0.33–2.95)	0.983	0.91	(0.29–2.85)	0.870
UPDRS 3	–	–	–	1.03	(1.00–1.07)	0.075

*BDI* Beck Depression Inventory, *UPDRS* Unified Parkinson's Disease Rating Scale, *UPSIT* University of Pennsylvania Smell Identification Test.

^a^Adjusted for age, gender.

^b^Adjusted for age, gender and UPDRS 3.

### Correlation between BDI-II scores and dysosmia, and between BDI-II scores and constipation in PD

**Figure 2:**
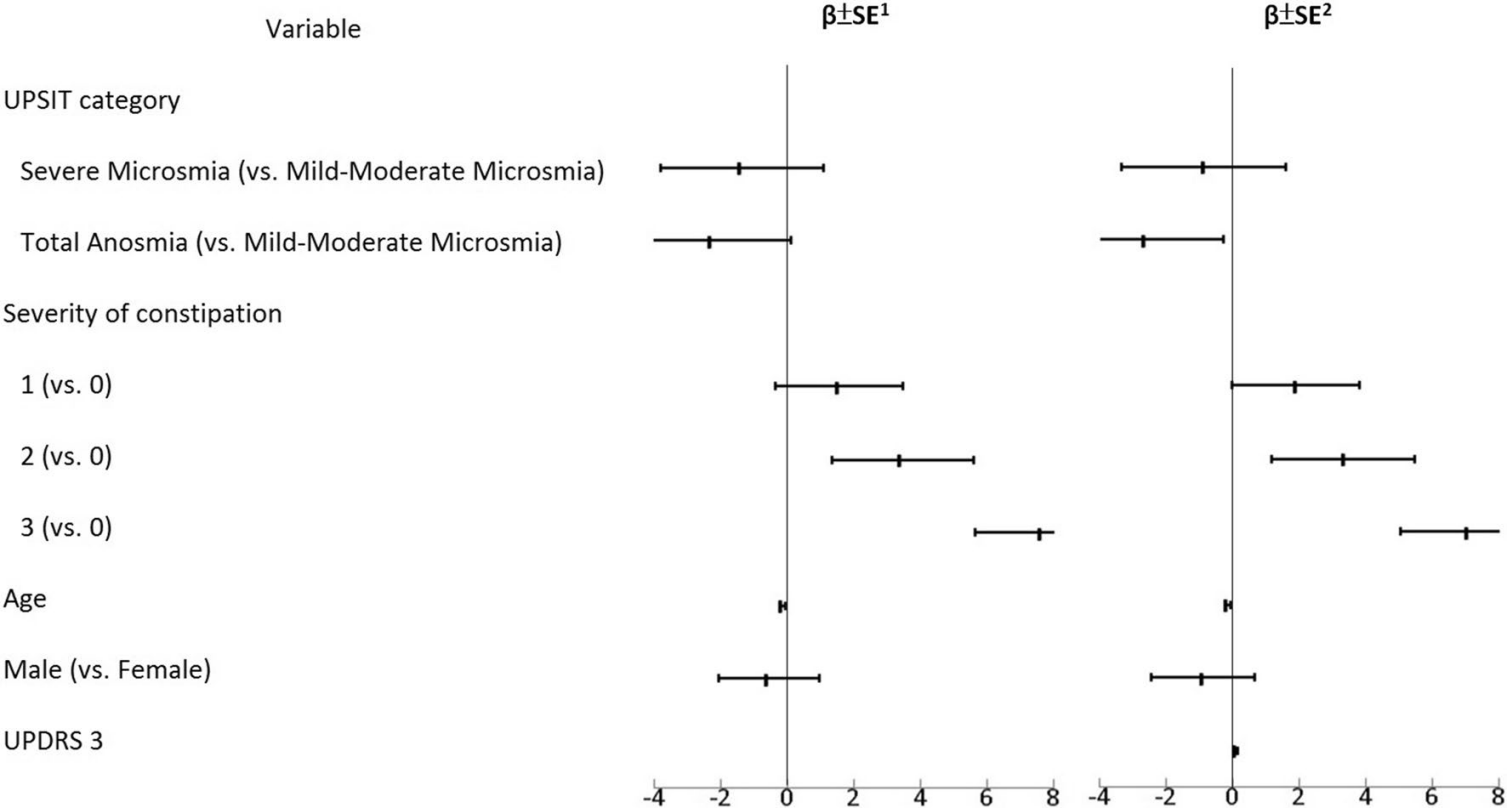
β coefficients of BDI-II scores associated with dysosmia and constipation. ^1^Adjusted for age and gender. ^2^Adjusted for age, gender and UPDRS 3. *BDI* Beck Depression Inventory, *UPDRS* Unified Parkinson's Disease Rating Scale, *UPSIT* University of Pennsylvania Smell Identification Test.

**Figure 3:**
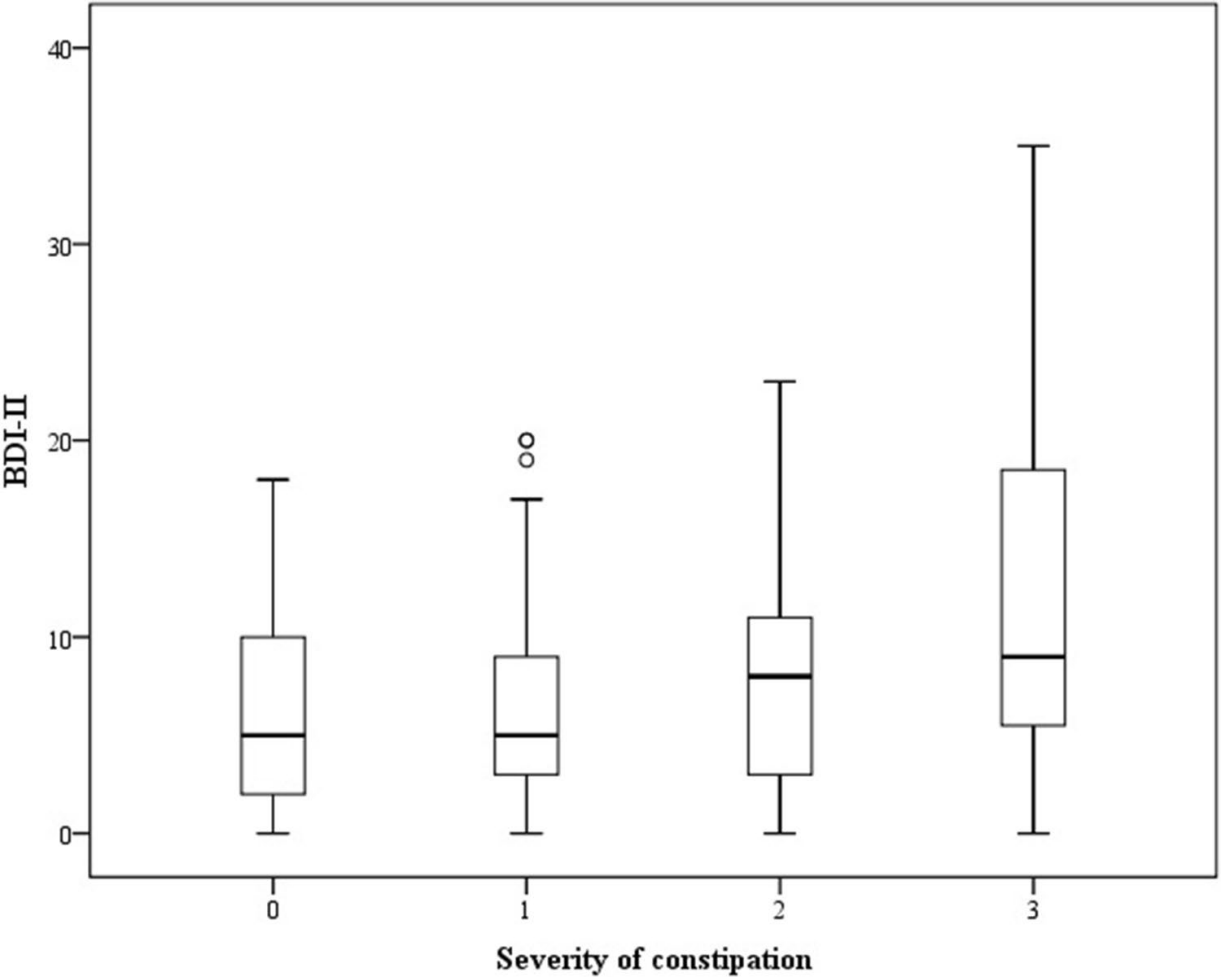
BDI-II scores in patients with different severities of constipation (grade 0, 1, 2, 3). *BDI* Beck Depression Inventory.

**Figure 4:**
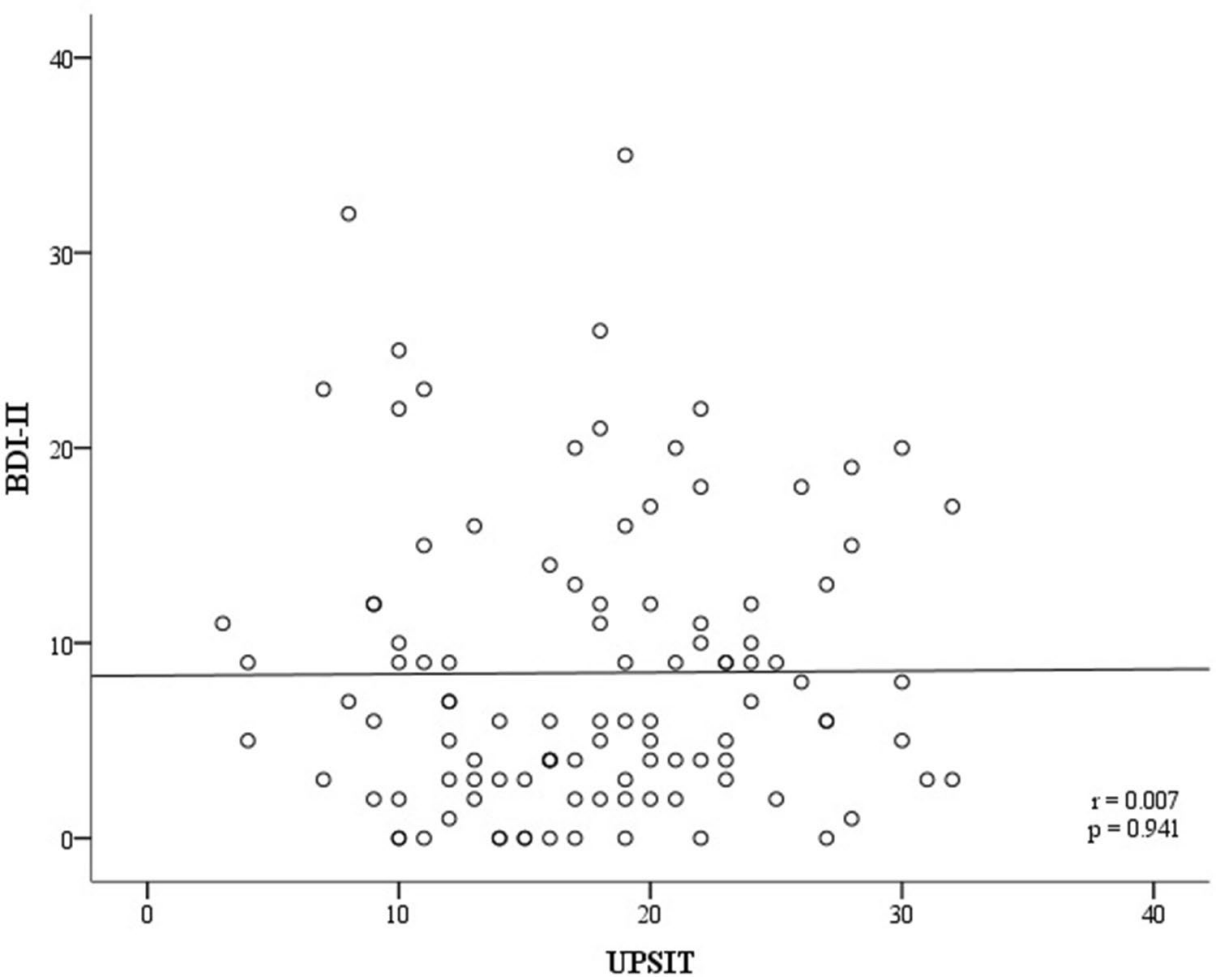
The correlation between BDI-II scores and the UPSIT scores. *BDI* Beck Depression Inventory, *UPSIT* University of Pennsylvania Smell Identification Test.

A linear regression model was constructed to examine the correlation between BDI-II scores and dysosmia, and the correlation between BDI-II scores and constipation. We adjusted the results by age, gender, and UPDRS-3. The results are shown in Table 3 and Fig. 2. We detected a significant positive correlation between BDI-II score and severe (grade 3) constipation (β ± SE 7.65 ± 2.02; p =  < 0.001, adjusted for age and gender; β ± SE 7.06 ± 2.04; p = 0.001, adjusted for age, gender, and UPDRS-3). A marginally significant positive correlation between BDI-II scores and motor function (UPDRS 3) was detected (β ± SE 0.09 ± 0.05; p = 0.064). However, there was no significant correlation between BDI-II scores and olfactory dysfunction. We illustrated the BDI-II scores of different severities of constipation in a box-plot (see Fig. 3) and the association between BDI-II and UPSIT scores in a scatter plot (see Fig. 4).

**Table 3:** β coefficients of BDI-II scores associated with dysosmia and constipation.

Variable	β ± SE^a^	P-value	β ± SE^b^	P-value
**UPSIT category**				
Mild-moderate microsmia vs. severe microsmia	− 1.36 ± 2.46	0.583	− 0.87 ± 2.47	0.726
Mild-moderate microsmia vs. total anosmia	− 2.29 ± 2.38	0.337	− 2.66 ± 2.37	0.265
**Severity of constipation**				
0 vs. 1	1.55 ± 1.93	0.422	1.9 ± 1.92	0.326
0 vs. 2	3.45 ± 2.13	0.108	3.33 ± 2.15	0.125
0 vs. 3	7.65 ± 2.02	< 0.001	7.06 ± 2.04	0.001
Age	− 0.15 ± 0.09	0.083	− 0.16 ± 0.09	0.064
**Gender**				
Female vs. male	− 0.55 ± 1.52	0.718	− 0.9 ± 1.54	0.559
UPDRS 3	–	–	0.09 ± 0.05	0.064

*BDI* Beck Depression Inventory, *UPDRS* Unified Parkinson's Disease Rating Scale, *UPSIT* University of Pennsylvania Smell Identification Test.

^a^Adjusted for age and gender.

^b^Adjusted for age, gender and UPDRS 3.

## Discussion

In this study, we investigated whether depression in PD patients is closely related to either constipation or dysosmia. We hypothesized that the degree of depression in PD patients may be related to the severity of constipation rather than olfactory dysfunction. Our results showed a significant association between DP group and severe constipation. When we conducted the linear regression, we detected a significant positive correlation between BDI-II scores and severe constipation, suggesting that severe constipation is associated with depression in PD. The gastrointestinal spreading of α-syn to brainstem nuclei is possibly the main mechanism by which depression is caused, rather than by the involvement of the limbic system through the olfactory bulbs. Lewy body pathology initially involves the enteric plexus of the gastrointestinal tract and spreads centrally to the DMV, which could cause the prodromal non-motor symptom of constipation in PD patients^3,14–18^. Afterwards, the DMV is connected to the raphe nuclei and LC. As the disease progresses, depressive moods may occur because of Lewy body inclusions occurring in the LC and raphe nucleus^9,19^. A few studies discussed the relationship of constipation and depression in PD. Yu et al. recruited a total of 306 PD patients and reported that depression was significantly more severe in PD patients with constipation (constipation versus non-constipation groups, Hamilton Depression Rating Scale [HAMD] scores 11.00 [median quartile: 5.00–19.00 versus 7.00 [3.00– 12.00]; p < 0.001). They suggested that constipation and depression might share the mechanism related to 5-hydroxytryptamine (5-HT) depletion in the PD population^20^. Gan et al. enrolled 268 PD patients and also reported significantly higher HAMD scores in PD with constipation (constipation versus non-constipation groups, HAMD scores 15.37 ± 11.38versus 12.05 ± 10.03; p = 0.019)^21^. In our study, we also observed a similar phenomenon, and it could be related to the pathway of α-syn propagation and involvement of important nuclei in brainstem, which leads to depression.

We did not detect an association between the severity of olfactory dysfunction and depression in PD patients. Morley et al. enrolled 248 PD patients and tested their olfactory function based on UPSIT scores and assessed depression using the 15-item Geriatric Depression Scale (GDS) and Inventory of Depressive Symptomatology (IDS). They found that the mean scores on these mood scales did not significantly differ between patients with better or worse olfactory function^22^. Berendse et al. recruited 96 PD patients and also assessed them with the UPSIT and BDI scales. They reported a statistically significant negative correlation between the UPSIT and BDI scores and indicated that higher levels of depressive symptoms were associated with worse odor identification scores^23^. Therefore, the association of olfaction and depression showed discrepancies and still remains controversial. Different clinical assessment tools of olfactory function and depression in addition to relatively small sample size may lead to these discrepancies. For example, the severity of depression could be assessed by HAMD, GDS or IDS, and the olfactory function could be assessed by Sniffin’ Sticks or Hyposmia Rating Scale (HRS)^22,23^. In our study, dysosmia may not be related to depression in PD patients. We presumed that the spread of α-syn from the olfactory bulb may spare some nuclei possibly related to depression in the brainstem, such as the raphe nucleus.

It is not surprising that a higher score on the UPDRS part 3 (motor function) was related to a higher BDI-II score, and we detected a marginally significant correlation between them. Worse motor function may also lead to larger psychosocial stress and may be explained by Braak staging. Non-motor symptoms, such as depression, may occur before motor symptoms because the Lewy body inclusions affect brainstem nuclei prior to affecting the substantia nigra. As the disease progresses, the propagation of α-syn eventually involves the substantia nigra and leads to typical manifestations of bradykinesia, rigidity, and tremors.

Non-motor subtypes of PD have been proposed in recent years based on the pathological evidence and recognized pathophysiological processes. Sauerbier et al. illustrated the current concepts of non-motor subtypes: (1) the brainstem phenotype, which represents the spread of Lewy bodies throughout the brainstem route or sometimes the olfactory route and presents with late onset hyposmia and prominent sleep and/or autonomic symptoms; (2) the limbic phenotype, in which spreading uses the olfactory route and presents with prominent anosmia initially, eventually leading to depression, fatigue, pain and/or weight loss; and (3) the cognitive phenotype, which represents the late onset pattern of Lewy body deposition in the neocortex and is characteristic of mild cognitive impairment, dementia and/or apathy^24^. The results of our study did not support the proposed concept that depression in PD patients is caused by the involvement of the limbic cortex through the olfactory route. Constipation, rather than dysosmia, seemed to be associated with depression in our PD patients. We speculated that depression may be closely relevant to brainstem nuclei rather than to the limbic pathway in our PD patients.

To our knowledge, there are few studies that have investigated the relationships between depression and constipation and dysosmia in patients with PD. Our study was an analytical cross-sectional study, and we collected data from only 106 patients in a single medical center. Our sample size was relatively small, and the patients were not equally distributed in the DP and NDP groups. Besides, there were many factors, such as psychosocial stress, genetics, pain, previous depression, and changes in neurotransmitter availability and function, that might influence depression in patients with PD^7^. Therefore, there may be some confounding factors for which we could not thoroughly control. Furthermore, we assessed dysosmia and constipation severities in our patients only once. Although we had tried to avoid biases during the assessments, there might be some conditions, such as acute sinusitis leading to hyposmia, short-term medications leading to constipation (opioid, antihistamine, or antimuscarinic agents), that we could not exclude. Of note, we found that most of our patients in the DP group did not use any antidepressants. PD patients may not be willing to mention their depressive mood to their doctors, or doctors may ignore mood symptoms because PD used to be considered a motor neurodegenerative disease. Only three patients took agomelatine under the indication of depression or insomnia. Agomelatine is a melatonin receptor (MT1/MT2) agonist plus a serotonin (5HT)-2C antagonist, which can sometimes cause constipation (1–10%). In consideration of other offending drugs possibly causing constipation, we found there were six patients taking anti-cholinergic/anti-muscarinic agents for their motor or urinary symptoms, but these patients did not have severe degree of constipation (see Supplementary Table S1 online).

In conclusion, our study investigated the clinical characteristics of PD with and without depression. PD patients with depression had more severe constipation and motor symptoms. The results were compatible with the pathway of α-syn propagation from the gastrointestinal tract to brainstem nuclei. Inclusions of the enteric plexus of the gastrointestinal tract and DMV initially cause constipation in PD patients. They then spread to the raphe nuclei and locus coeruleus, which lead to depressive mood. Involvement of the substantia nigra subsequently leads to motor symptoms. However, in our study, olfactory dysfunction appeared to not be relevant to depression. We speculated that depression in PD patients may be closely relevant to brainstem nuclei rather than to the limbic pathway. However, this is a clinical observation study, and further investigations are needed.

## Supplementary information

Supplementary Information 1.

## Data Availability

The datasets generated during and/or analyzed during the current study are available from the corresponding author.
