# Supplementary material for: Is there a close association of depression with either constipation or dysosmia in Parkinson’s disease?
Source: Sci Rep. 2020 Sep 23;10:15476. doi: 10.1038/s41598-020-72381-0 (PMC7511293; doi:10.1038/s41598-020-72381-0)
Supplement: Supplementary file 1 — Supplementary Information 1. [file 41598_2020_72381_MOESM1_ESM.pdf]

Is there a close association of depression with either constipation or dysosmia in Parkinson's disease?

Ting-Ya Chang, MD[1], Yi-Huei Chen, BS[2], Ming-Hong Chang, MD[1,*], and Ching-Heng Lin, PhD[2]

[1] Taichung Veterans General Hospital, Department of Neurology

[2] Taichung Veterans General Hospital, Department of Medical Education and Research

* corresponding author: cmh50@ms10.hinet.net

No.	Gender	Age (years)	Severity of constipation	BDI-II	Antidepressants	Other medications may cause constipation
**DP-1**	F	80	3	23	-	-
**DP-2**	M	65	3	25	-	-
**DP-3**	M	55	1	17	-	-
**DP-4**	M	57	3	17	-	Benzhexol 1 mg BID
**DP-5**	F	74	3	26	-	-
**DP-6**	M	68	3	15	-	-
**DP-7**	F	74	3	32	-	-
**DP-8**	F	49	1	20	-	-
**DP-9**	M	63	0	15	-	Biperidin 1 mg BID
**DP-10**	M	56	0	16	-	-
**DP-11**	M	73	3	20	-	-
**DP-12**	F	54	2	23	-	Oxybutynin 5 mg QD
**DP-13**	F	53	1	19	Agomelatine 25 mg HS	-
**DP-14**	M	72	3	22	-	-
**DP-15**	M	56	2	21	-	-
**DP-16**	M	52	3	35	-	-
**DP-17**	M	68	1	20	-	-
**DP-18**	F	57	1	16	-	Biperidin 1 mg BID
**DP-19**	F	44	0	18	Agomelatine 25 mg HS	-
**DP-20**	M	61	2	22	Agomelatine 25 mg HS	-
**DP-21**	F	74	0	14	-	-
**DP-22**	M	68	2	18	-	Benzhexol 1 mg BID

Supplementary table S1. List of antidepressants for PD patients with depression (DP group, BDI$\geqq$14). Abbreviations: BDI=Beck Depression Inventory, DP group=Depressive group. F=female, M=male, HS=at bedtime, QD=once daily, BID=twice daily, TID=thrice daily.
